# Imaging Identification and Prognosis of the Distal Internal Carotid Artery With Near and Complete Occlusion After Recanalization

**DOI:** 10.3389/fneur.2020.630028

**Published:** 2021-02-05

**Authors:** Tao Sun, Chao Wang, Mengtao Han, Fei Wang, Yiming He, Yunyan Wang, Xingang Li, Donghai Wang

**Affiliations:** ^1^Department of Neurosurgery, Qilu Hospital, Cheeloo College of Medicine, Shandong University, Jinan, China; ^2^Shandong Key Laboratory of Brain Function Remodeling, Cheeloo College of Medicine, Shandong University, Jinan, China; ^3^Dezhou City People's Hospital, Dezhou, China; ^4^Department of Neurosurgery, Binzhou Medical University Hospital, Binzhou, China

**Keywords:** hybrid recanalization, internal carotid artery, near occlusion, occlusion, low-perfusion narrowness

## Abstract

**Background and Purpose:** Previous studies have mainly focused on treatment strategies and clinical outcomes for internal carotid artery near occlusion (ICANO) and internal carotid artery complete occlusion (ICACO). However, reports on the morphological changes of distal internal carotid artery (ICA) after recanalization are scarce. This study aimed at illustrating identifying features, assessing prognosis of the distal ICA after recanalization, and exploring best practices for treatment for ICANO and ICACO.

**Materials and Methods:** We retrospectively studied the clinical characteristics of 57 patients with ICANO or ICACO who underwent surgical recanalization. The clinical data, angiographic morphology, technical successful rate, perioperative complications, and the lumen changes of distal ICA before and after successful recanalization were analyzed.

**Results:** Fifty-two patients who achieved successfully recanalization were studied. Based on the postoperative lumen diameter changes in the distal ICA, 19 cases were classified as distal-dilatation and the remaining 33 as distal-narrowness. Patients in the distal-narrowness group mostly had ICACO (21.1 vs. 54.5%) and were men (68.4 vs. 93.9%). In the distal-narrowness group, the lumen of the distal ICA recovered to normal in 32 of the 33 patients during the follow-up period. Of the 32 patients reviewed, the ICA of 28 patients dilated back to normal after 1 week of surgery; the ICA of remaining patients 4 dilated 2 weeks postoperatively.

**Conclusions:** Narrowness of the distal ICA after hybrid recanalization was more prevalent in male patients with ICACO. Homogeneous stenosis of the whole course of the distal ICA is a low-perfusion narrowness which does not require intervention and will spontaneously recover after successful recanalization with an increase in the forward flow.

## Introduction

Near occlusion of internal carotid artery (ICANO), also known as pseudo-occlusion, subtotal occlusion, string sign of the internal carotid artery (ICA), describes a phenomenon of an obvious reduction of the artery diameter beyond the stenotic lesion in an ICA with severe stenosis (1, 2). It represents a critical stenotic state before ICA severe stenosis progresses to complete occlusion (ICACO). Follow-up in the drug-treated group showed that about 40% of near occlusion of the ICA progressed to total occlusion within 12 months (3, 4). Therefore, the diameters decrease and even collapse of distal lumens can be discovered in both ICANO and ICACO.

Drug therapy is still the preferred treatment in treating carotid ICANO and ICACO, but ischemic symptoms refractory to medical therapy are an indication for revascularization (5–7). Both carotid endarterectomy (CEA) and endovascular intervention (EI) with a proximal embolic protection device have achieved satisfactory results for ICANO (8). As for ICACO, hybrid surgery is a feasible and effective surgical method, which combined CEA, immediate intraoperative angiography, and EI in a hybrid operating suite (7, 9). Recent studies have reported that compared with EI, hybrid surgery has a higher success rate of recanalization and less complication for ICACO (5, 6, 10). In clinical practice, we discovered that two changes existed in the distal ICA after surgical or hybrid recanalization: distal-dilatation and distal-narrowness. In this study, we retrospectively analyzed the angiographic characteristics, illustrated identifying features of the two lesions, and we investigated the postoperative short-term change in the diameter of the distal ICA to explore best practices for treatment of ICANO and ICACO.

## Materials and Methods

### Patients and Materials

We retrospectively studied the medical records of patients with symptomatic ICANO or chronic ICACO treated at our institution between December 2015 and April 2020. ICANO is diagnosed if patients meet the following criteria: (1) delayed imaging of the ICA compared with that of the external carotid artery (ECA); (2) contrast agent filled the intracranial branches of the ICA via collateral circulation, usually via the ophthalmic artery; (3) obviously reduced diameter of the ICA (2). Chronic ICACO was defined as total occlusion of the ICA for at least 4 weeks seen on an angiogram (11).

We reviewed demographic characteristics, such as age, sex, history of disease (hypertension, diabetes mellitus, diabetes, and ischemic heart disease), smoking status and alcohol drinking status. The detail about symptomatology (transient ischemic attack, stroke), treatment modalities and perioperative complications were collected. And the level of total cholesterol, homocysteine (hCY), low density lipoprotein (LDL), and high density lipoprotein (HDL) were recorded.

**Figure 1:**
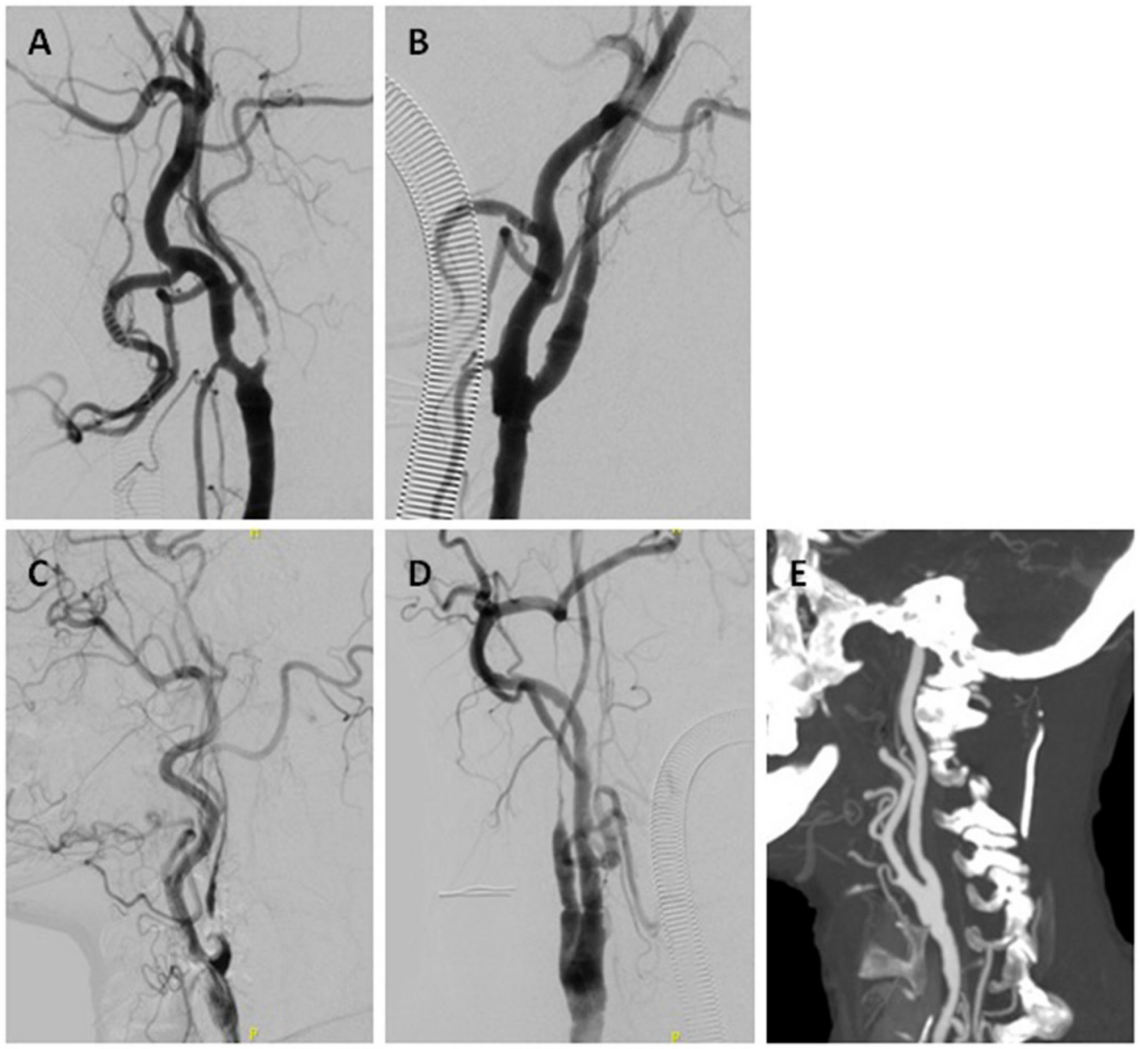
Two cases of ICANO with hybrid recanalization. Case-1 **(A,B)** A 66-year-old woman complaining of TIA 2 months was hospitalized. **(A)** Digital subtraction angiogram (DSA) before the hybrid surgery revealed a left ICANO. **(B)** Immediate angiogram of the left ICA after recanalization showed distal-dilatation. CASE-2 **(C–E)** A 71-year-old man complaining of glossolalia and left limbs weakness 20 days was hospitalized. **(C)** Preoperative DSA revealed a right ICANO. **(D)** Immediate angiogram of right ICA after recanalization showed distal-narrowness. **(E)** Computed tomography angiogram (CTA) performed 1 week postoperatively showed dilated right ICA.

We classified the post-recanalization findings of the ICA beyond the stenotic lesion into distal-dilatation (Figure 1B) and distal-narrowness groups (Figure 1D). Referring to the study of Rothwell et al. (12), the former was defined as the cases with an ICA/common carotid artery (CCA) ratio of ≥0.42 and the latter with a ratio <0.42. Patients who performed a deployment of stents due to intraoperative carotid segment dissection were outside the scope of the study, but included the calculation of the technical success rate. The morphological changes in the distal ICA lumen were followed up with computed tomographic angiography (CTA), magnetic resonance angiography (MRA), or digital subtraction angiography (DSA).

The study was approved by the hospital's ethics committee. Informed consent was obtained from all patients.

### Surgical or Hybrid Treatment

All patients daily received dual-antiplatelet medication (aspirin 100 mg and clopidogrel 75 mg) for at least 3 days before the procedure. The procedure was performed in a hybrid operating room and general anesthesia was administered in all patients. An incision was made in the anterior border of the sternocleidomastoid muscle. After the patient was fully heparinized with 125 IU of heparin per kg of body weight intravenously, carotid bifurcation was exposed and then isolated by sequentially cross-clamping the internal, external, and common carotid arteries. The artery is opened and the plaque removed. A mechanical thrombectomy was cautiously attempted with a 4F Fogarty embolectomy balloon catheter, especially in ICACO cases. The ICA was subsequently sutured. Next, a immediate intraoperative carotid angiography was performed via a right femoral puncture using the Seldinger technique. If forward flow of the ICA recovered, we sutured the wound. If not, a 6F catheter was used to access the proximal occlusion site. Then a 0.014-inch microguidewire and a microcatheter cross the occlusion under the guidance of the roadmap. A balloon-mounted stent angioplasty was performed in the distal ICA, usually in the petrous/cavernous segment. No stent was implanted in carotid segment, unless intraoperative maneuvers caused carotid segment dissection.

### Statistical Analysis

Normally distributed quantitative data was expressed as the mean ± standard deviation, and was analyzed by the Student's *t*-test. Non-normally distributed quantitative data was expressed as the median and interquartile range, and was analyzed by the Mann-Whitney *U* test. The qualitative data was expressed as percentage, and was analyzed using the chi-square test or the Fisher exact test. Logistic regression was used to evaluate effect of relevant risk factors on distal-narrowness after recanalization. A *P* < 0.05 was considered significant. SPSS software was used for data analysis.

## Results

Among the 57 patients with ICANO or ICACO who received hybrid treatment, a total of 53 achieved successful recanalization. Among these successful cases, a patient who performed a deployment of stents due to intraoperative carotid segment dissection was excluded from this study. The clinical characteristics, procedure, and perioperative complications of the patients in these two lesions are summarized in Table 1. The distal-dilatation and distal-narrowness groups consisted of 19 and 33 patients, respectively. The mean age of the patients was 63.74 ± 7.53 years in the distal-dilatation group and 62.21 ± 6.64 years in the distal-narrowness group (*P* = 0.45). Patients in the distal-narrowness group were more likely to have ICACO (21.1% for distal-dilatation vs. 54.5% for distal-narrowness group; *P* = 0.02) and were males (68.4% for distal-dilatation vs. 93.9% for distal-narrowness group; *P* = 0.04).

**Table 1:** Patient characteristics, procedure and perioperative complication.

	**Distal-dilatation**	**Distal-narrowness**	***P*-value (<0.05)**
No. patients	19	33	
**Patient characteristics**			
Age	63.74 ± 7.53	62.21 ± 6.64	0.45
Sex male	14 (68.4%)	30 (93.9%)	0.04
Symptom			0.08
TIA Stroke	11 (57.9%) 8 (42.1%)	11 (33.3%) 22 (57.7%)	
Duration from initial symptom to recanalization, median (IQR), month	2 (1–2)	3 (1–10.5)	0.07
ICACO	4 (21.1%)	18 (54.5%)	0.02
Hypertension	16 (84.2%)	25 (75.8%)	0.73
Diabetes mellitus	5 (26.3%)	13 (39.4%)	0.34
Ischemic heart disease	3 (15.8%)	4 (12.1%)	0.70
History of smoking	9 (47.4%)	12 (36.4%)	0.44
Total cholesterol (mmol/L)	3.49 ± 0.80	3.49 ± 0.90	1.00
hCY (μmol/L)	15.06 ± 6.23	15.07 ± 6.19	1.00
LDL (mmol/L)	1.90 ± 0.75	2.04 ± 0.70	0.50
HDL (mmol/L)	1.12 ± 0.25	1.02 ± 0.22	0.16
**Procedure**			
Mechanical thrombectomy	2 (10.5%)	6 (18.2%)	0.74
Balloon and stent angioplasty in the petrous/cavernous segment	4 (21.1%)	9 (27.3%)	0.87
**Perioperative complication**			
Cardiovascular events	0	2 (6.1%)	0.53
CHS	1 (5.3%)	0	0.37
Intracranial hematoma	1 (5.3%)	0	0.37

*TIA, transient ischemic attack; IQR, indicates interquartile range; ICACO, internal carotid artery complete occlusion; hCY, homocysteine; LDL, low density lipoprotein; HDL, high density lipoprotein; CHS, cerebral hyperperfusion syndrome*.

There were no significant differences in terms of symptom, duration from initial symptom, hypertension, diabetes mellitus, ischemic heart disease, smoking history, status of alcohol drinking, serum total cholesterol, hCY, LDL, HDL level, procedure and perioperative complications between the two groups. In perioperative period, two patients showed an increase in the myocardial enzyme. One ICANO patient with distal-dilatation experienced an perioperative intracranial hematoma due to cerebral hyperperfusion syndrome (CHS), and recovered conservatively.

**Figure 2:**
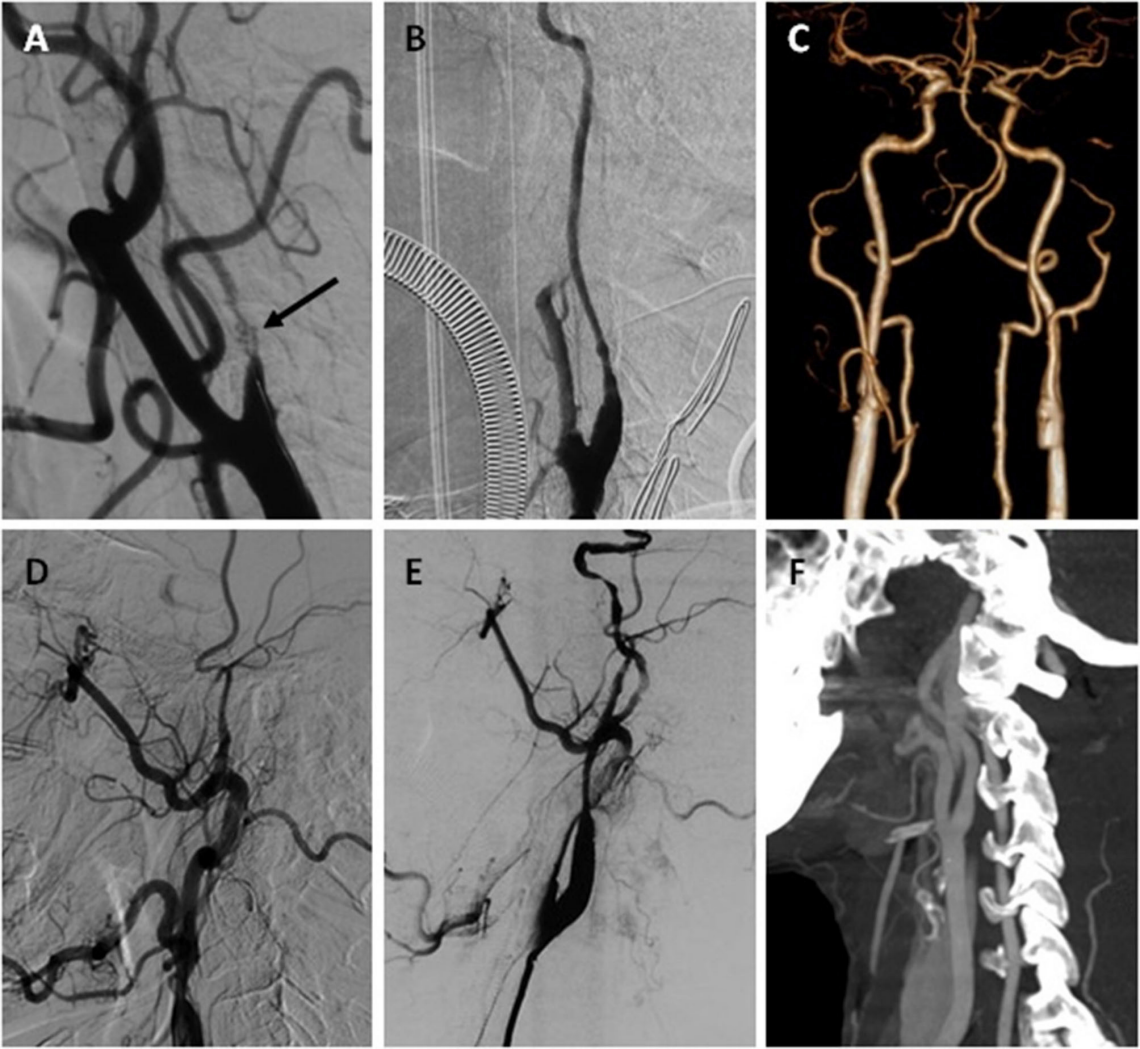
One ICANO and one ICACO with hybrid recanalization. Case-3 **(A–C)** A 56-year-old man complaining about TIA 2 years was hospitalized. **(A)** DSA before the hybrid surgery revealed a left ICANO with total occlusion and recanalization, which is characterized by multiple lumens (arrow). **(B)** Immediate angiogram of the left ICA after recanalization showed distal-narrowness. **(C)** CTA performed 1 week postoperatively showed a dilated left ICA. Case-4 **(D–F)** A 59-year-old man complaining of expressive aphasia 1 month was hospitalized. **(D)** Preoperative DSA revealed a left ICACO. **(E)** Immediate angiogram of left ICA after recanalization showed distal-narrowness. **(F)** (CTA) performed 1 week postoperatively showed dilated left ICA.

In the distal-narrowness group, lumen of the distal ICA dilated to normal in 32 of the 33 patients in the follow-up period about 1–2 weeks (Figures 1, 2). However, one case (3.03%) still showed distal-narrowness 1 month later.

The logistic regression analysis revealed that factors significantly associated with distal-narrowness were ICACO and male (odds ratio 5.30, 95% confidence interval: 1.26–22.29, *P* = 0.02; odds ratio 8.83, 95% confidence interval: 1.33–58.81, *P* = 0.02, Table 2).

**Table 2:** Results of logistic regression analysis for distal-narrowness group.

	**Odds ratio**	**95% Confidence interval**		***P*-value**
		**Lower**	**Upper**	
ICACO	5.30	1.26	22.29	0.02
Men	8.83	1.33	58.81	0.02

## Discussion

Carotid atherosclerotic stenosis is a common disease and is closely related to ischemic cerebrovascular events. Internal carotid artery near occlusion (ICANO), also known as pseudo-occlusion, subtotal occlusion or string sign of ICA, describes a phenomenon of an obvious reduction of the artery diameter beyond the stenotic lesion in an ICA with severe stenosis (1, 2). On angiograms, the distal ICA becomes narrow or even collapsed. A major collapse of the ICA described as the “string sign,” “slim sign,” “small distal ICA,” or “post-stenotic narrowing,” (13–15) represents the critical state before internal carotid artery stenosis progresses to total occlusion. Ultimately, 40% of the near occlusion of ICA progress to total occlusion within 12 months (3, 4). The present definition of chronic ICACO is controversial. The minimum time for chronic total occlusion of the ICA should be at least 4 weeks and possibly even more than 3 months (11). Hybrid surgery is a newly-developing treatment which combines CEA, immediate intraoperative angiography, and EI (9). In a hybrid operation, we can get intraoperative angiograms after CEA, and instantly decide whether to perform mechanical thrombectomy and/or endovascular angioplasty, which raises the success rate of recanalization and is especially suitable for the treatment of ICACO. Li et al. (6) reported that hybrid surgery have a higher success rate of recanalization than EI in patients with ICACO (88.2 vs. 53.3%; *P* = 0.05). Our success rate for cases with ICACO is 85.19% (23/27), and that for cases with ICANO is 96.77% (30/31). Therefore, hybrid surgery is effective for recanalization of patients with ICANO and ICACO.

On intraoperative angiograms after successful recanalization, we discover that the distal ICA beyond a critical stenosis shows two states: (1) distal ICA with an approximate normal diameter; and (2) distal ICA with homogeneous narrowness. It is difficult to confirm normal diameter of ICA, but the lower limit of normal ICA/CCA ratio is 0.42 by studying angiograms of carotid arteries with 0–49% stenosis (12). Therefore, we define the cases with an ICA/CCA ratio of ≥0.42 as distal-dilatation corresponding to the former state and the ones with an ICA/CCA ratio of <0.42 as distal-narrowness corresponding to the latter state. Among our 52 patients with successful recanalization, 19 (36.5%) cases were distal-dilatation and 33 (63.5%) cases were distal-narrowness.

Early carotid atherosclerosis only causes slight stenosis, with a normal diameter of the distal ICA. As the stenosis progressively becomes more severe and exceeds a critical degree, a further deterioration in stenosis will result in a decrease of forward flow, and the diameter of the ICA beyond the stenosis progressively begins to decrease. By sacrificing the diameter, the distal ICA makes wall shear stress recover a baseline of 15~20 dyn/cm^2^, which not only provides effective support in stabilizing blood vessel wall, but also maintains adequate perfusion. When blood vessels have a chronically low wall shear stress for a long time, vascular remodeling will happen by accumulation and differentiation of the intimal cell (16). Irace et al. (17) also reported that intima-media thickening is significantly associated with low wall shear stress. Meanwhile, reduction of shear stress can alter the endothelial production of bioactive molecules related to vascular tone, which may achieve through regulating the expression of gene (18). As a result, the blood vessel wall incrassates, the lumen becomes narrow, and the distal ICA takes a longer time to reverse the vascular remodeling after recanalization. Therefore, ICACO is more likely to be distal-narrowness because of a longer period of distal ICA flow impairment than ICANO.

In our 30 patients with ICANO, six patients underwent spontaneous recanalization of atheromatous chronic ICA occlusion. ICANO with total occlusion and recanalization is likely to show multiple lumens caused by large neovascular channels on angiography (Figure 2A) (19). Although spontaneous recanalization partly causes recovery of the flow, there is less improvement in the forward flow of the distal ICA. Moreover, ICANO with total occlusion and recanalization may represent a longer course of illness and is more likely to show distal-narrowness.

The incidence of postoperative hyperperfusion syndrome was about 0.2–18.9% (20), but once it happens, it caused serious consequences (21). In perioperative complications, one distal-dilatation patient with ICANO had a CHS and suffered from subsequent intracranial hematoma caused by CHS in the perioperative period. We speculate that a sudden increase of blood flow in distal-dilatation ICA may partially cause this malignant event. Hayakawa et al. (22) reported that staged angioplasty was effective to avoid CHS after carotid revascularization. The process from narrowness to dilatation of the distal ICA after CEA actually can be seen as natural staged angioplasty and may be helpful to prevent CHS. The process from narrowness to dilatation of the distal ICA after CEA actually can be seen as natural staged angioplasty and may be helpful to prevent CHS. Therefore, we think that distal-dilatation patients may require more strict blood pressure management and more intensive medical monitor to prevent and early find the occurrence of CHS after recanalization. CT perfusion (CTP), single-photon emission computed tomography or transcranial Doppler (TCD) examinations to monitor the perfusion of brain may be helpful for further research to illustrate the significance of distal-narrowness for avoidance of CHS.

In EI and some hybrid surgeries for ICACO, the distal ICA can be dilated by reconstructing with balloons and stents (23, 24). We occasionally performed a balloon and stent angioplasty distally, specifically in the petrous/cavernous segment, so reduced diameter of the distal ICA still existed in our distal-narrowness group. However, we discovered that the distal ICA was dilated in 32 of 33 patients in the follow-up examination. The average recovery time was 1.125 week. Partial cases were shown in Figures 1, 2. We think that the restoration of the vessel diameter is an adaptation to increased postoperative blood flow, and slowly progressed by remodeling the vessel wall. However, the ICA/CCA ratio of one patient was still <0.42. Distal ICA of the patient showed local stenosis of the siphonage segment after 1 month, but the lesion was not observed on intraoperative angiography. We assume that homogeneous narrowness of the distal ICA covered up local atherosclerotic stenosis, and the existence of distal local stenosis prevents the flow of ICA from increasing in these cases.

Although the balloon-mounted stent angioplasty can achieve distal-dilatation, we think that the homogeneous stenosis of the whole course of distal ICA is a low-perfusion narrowness, which does not require intervention and will shortly expand with an increase in the forward flow after successful recanalization. However, unobserved and unperformed tandem lesion in intraoperative angiography will prevent distal ICA from recovering, so regular follow-up is needed in patients with distal-narrowness ICA.

There are limitations deserving mention in our study. Firstly, the sample size in the present study was relatively small. Secondly, our study was a retrospective one. Thirdly, the lack of data of postoperative cerebral perfusion limited assessment of significance of distal-narrowness in avoiding CHS. Finally, histological data on distal vessel wall obtained by high resolution magnetic resonance (HR-MRI), optical coherence tomography and carotid endarterectomy may be helpful to clarify the potential mechanisms about the changes in the distal ICA.

## Conclusions

In summary, hybrid recanalization is an effective method for patients with ICANO or ICACO. The diameter changes in the distal ICAs after recanalization were classified into two groups, a distal-dilatation group and distal-narrowness group. Patients in the distal-narrowness group were more likely to have ICACO and to be males. The homogeneous stenosis of the whole course of distal ICA is a low-perfusion narrowness, which does not require intervention, and will recover spontaneously after successful recanalization with an increase in the forward flow.

## Data Availability Statement

The raw data supporting the conclusions of this article will be made available by the authors, without undue reservation.

## Ethics Statement

The studies involving human participants were reviewed and approved by the Ethics Committee of the Qilu Hospital, Shandong University. The patients/participants provided their written informed consent to participate in this study.

## Author Contributions

XL and DW established the study idea, designed the manuscript structure. TS, MH, YH, and FW were responsible for the data collection. TS and CW analyzed the data and wrote the manuscript. XL, DW, and YW modified and revised the manuscript. All authors have read and approved the final version of the manuscript.

## Conflict of Interest

The authors declare that the research was conducted in the absence of any commercial or financial relationships that could be construed as a potential conflict of interest.

## Abbreviations

ICANO: internal carotid artery near occlusion
ICACO: internal carotid artery complete occlusion
ICA: internal carotid artery
CEA: carotid endarterectomy
EI: endovascular intervention
CCA: common carotid artery
CTA: computed tomographic angiography
DSA: digital subtraction angiography
CHS: cerebral hyperperfusion syndrome
TIA: transient ischemic attack.
